# Supplementary material for: Economic Evaluation of Oral Cancer Screening Programs: Review of Outcomes and Study Designs
Source: Healthcare (Basel). 2023 Apr 21;11(8):1198. doi: 10.3390/healthcare11081198 (PMC10138408; doi:10.3390/healthcare11081198)
Supplement: Supplementary file 1 [file healthcare-11-01198-s001.zip › Supplementary Material Table S1.pdf]

Supplementary Material Table S1: Search terms examples

COCHRANE Search Strategy for Systematic Review of Economic Evaluation in Oral Cancer and OPMD screening

Oral cancer	Mouth-neoplasm or Gingival-neoplasm or Lip-neoplasm or Tongue-neoplasm or squamous cell carcinoma* or buccal mucosa or oral carcinoma
OPMD	Leukoplakia-oral or premalig* or pre-malig* oral or oral potential* malig* or oral dysplasia or oral lesion*
screening	Screening or Diagnosis or oral examination or diagnostic test
Economic evaluation	Economic or Cost or Cost effectiveness or Cost utility or Cost benefit
Exclusion	Not skin or breast or lung or anal or viral or colorectal or cervical or liver or gastric

Example of search process using Boolean operators in COCHRANE

#1	Mouth-neoplasm or Gingival-neoplasm or Lip-neoplasm or Tongue-neoplasm or squamous cell carcinoma* or buccal mucosa or oral-carcinoma
#2	Leukoplakia-oral or oral premalig* or pre-malig* oral or oral potential* malig* or oral dysplasia or oral lesion*
#3	#1or#2
#4	Screening or Diagnosis or oral-examination or visual examination or diagnostic test
#5	#3 and #4
#6	Economic or Cost or Cost effectiveness or Cost utility or Cost benefit
#7	#5 and #6
#8	#7 with Cochrane Library publication date Between Jan 2000 and Jan 2019
#9	#7 and adult
#10	#9 not (lung or skin or stroke or diabetes or pain or schizo*)
