# Supplementary material for: Economic Evaluation of Oral Cancer Screening Programs: Review of Outcomes and Study Designs
Source: Healthcare (Basel). 2023 Apr 21;11(8):1198. doi: 10.3390/healthcare11081198 (PMC10138408; doi:10.3390/healthcare11081198)
Supplement: Supplementary file 1 [file healthcare-11-01198-s001.zip › Supplementary Material Table S2.pdf]

Supplementary Material Table S2: Excluded full-text articles

Study	Reason
Vokó Z, Túri G, Zsólyom A. A szájüregi szűrés költséghatékonysága Magyarországon. Cost-effectiveness of oral cancer screening in Hungary. Orv Hetil. 2016 Jul;157(29):1161-70	Unable to obtain a valid translated copy
Birur NP, Patrick S, Bajaj S, Raghavan S, Suresh A, Sunny SP, et al. A Novel Mobile Health Approach to Early Diagnosis of Oral Cancer. The journal of contemporary dental practice. 2018;19(9):1122-8.	No economic evaluation
Balevi B. Assessing the usefulness of three adjunctive diagnostic devices for oral cancer screening: a probabilistic approach. Community dentistry and oral epidemiology. 2011;39(2):171-6.	No economic evaluation
Brocklehurst P, Kujan O, O'Malley LA, Ogden G, Shepherd S, Glenny AM. Screening programmes for the early detection and prevention of oral cancer. Cochrane Database of Systematic Reviews. 2013(11).	Systematic review
Cariati P, Cabello-Serrano A, Perez-de Perceval-Tara M, Monsalve-Iglesias F, Martínez-Lara I. Oral and oropharyngeal squamous cell carcinoma in young adults: A retrospective study in Granada University Hospital. Medicina Oral, Patologia Oral Y Cirugia Bucal. 2017;22(6):e679-e85.	No economic evaluation
Chatterjee R, Gupta B, Bose S. Oral Screening for Pre-cancerous Lesions Among Areca-nut Chewing Population from Rural India. Oral Health & Preventive Dentistry. 2015;13(6):509-14.	No economic evaluation
Cromwell I, Regier DA, Peacock SJ, Poh CF. Cost-Effectiveness Analysis of Using Loss of Heterozygosity to Manage Premalignant Oral Dysplasia in British Columbia, Canada. The oncologist. 2016;21(9):1099-106.	Genetic testing as a screening method
Eadie D, MacKintosh AM, MacAskill S, Brown A. Development and evaluation of an early detection intervention for mouth cancer using a mass media approach. British journal of cancer. 2009;101 Suppl 2:S73-9.	No economic evaluation
Gandhi S, Lata J, Gandhi N. Fine needle aspiration cytology: a diagnostic aid for oral lesions. Journal of Oral & Maxillofacial Surgery (02782391). 2011;69(6):1668-77.	Needle aspiration cytology
Gonzalez Segura I, Secchi D, Carrica A, Barello R, Arbelo D, Burgos A, et al. Exfoliative cytology as a tool for monitoring pre-malignant and malignant lesions based on combined stains and morphometry techniques. Journal Of Oral Pathology & Medicine: Official Publication Of The International Association Of Oral Pathologists And The American Academy Of Oral Pathology. 2015;44(3):178-84.	No economic evaluation
Hur C, Choi SE, Kong CY, Wang G-Q, Xu H, Polydorides AD, et al. High-resolution microendoscopy for esophageal cancer screening in China: A cost-effectiveness analysis. World Journal Of Gastroenterology. 2015;21(18):5513-23.	Microendoscopy in esophageal cancer
Jacobson JJ, Epstein JB, Eichmiller FC, Gibson TB, Carls GS, Vogtmann E, et al. The cost burden of oral, oral pharyngeal, and salivary gland cancers in three groups: commercial insurance, Medicare, and Medicaid. Head & Neck Oncology. 2012;4:15-.	No screening
Junaid M, Suhail A, Umer B, Nawaz A, Ikram M, Sobani Z-u-A, et al. Toluidine blue: yet another low cost method for screening oral cavity	No economic evaluation

tumour margins in third world countries. JPMA The Journal Of The Pakistan Medical Association. 2013;63(7):835-7.	
Lee MK, Dodson TB, Nalliah RP, Karimbux NY, Allareddy V. Nine-year trend analysis of hospitalizations attributed to oral and oropharyngeal cancers in the United States. Oral Surgery, Oral Medicine, Oral Pathology & Oral Radiology. 2014;118(1):47-67.	No screening
Lim K, Moles DR, Downer MC, Speight PM. Opportunistic screening for oral cancer and precancer in general dental practice: results of a demonstration study. British Dental Journal. 2003;194(9):497-502.	No economic evaluation
Nunn H, Lalli A, Fortune F, Croucher R. Oral cancer screening in the Bangladeshi community of Tower Hamlets: a social model. British journal of cancer. 2009;101 Suppl 2:S68-72.	No economic evaluation
Rahman MS, Ingole N, Roblyer D, Stepanek V, Richards-Kortum R, Gillenwater A, et al. Evaluation of a low-cost, portable imaging system for early detection of oral cancer. Head & neck oncology. 2010;2(1):10.	No economic evaluation
Sweeny L, Dean NR, Magnuson JS, Carroll WR, Clemons L, Rosenthal EL. Assessment of tissue autofluorescence and reflectance for oral cavity cancer screening. Otolaryngology--Head And Neck Surgery: Official Journal Of American Academy Of Otolaryngology-Head And Neck Surgery. 2011;145(6):956-60.	No economic evaluation
Uthoff RD, Song B, Sunny S, Patrick S, Suresh A, Kolur T, et al. Point-of-care, smartphone-based, dual-modality, dual-view, oral cancer screening device with neural network classification for low-resource communities. PloS one. 2018;13(12):e0207493.	No economic evaluation
Yan SK, Wei BJ, Lin ZY, Yang Y, Zhou ZT, Zhang WD. A metabonomic approach to the diagnosis of oral squamous cell carcinoma, oral lichen planus and oral leukoplakia. Oral oncology. 2008;44(5):477-83.	Metabonomics-based diagnostic approach
Younis RT, Hesse SV, Anand VK. Evaluation of the utility and cost-effectiveness of obtaining histopathologic diagnosis on all routine tonsillectomy specimens. The Laryngoscope. 2001;111(12):2166-9.	Histopathologic screening
Meregaglia M, Cairns J, Licitra L, and Bossi P. The use of intensive radiological assessments in routine surveillance after treatment for head and neck cancer: An economic evaluation. Eur J Cancer, 2018. 93: p. 89-98. DOI: 10.1016/j.ejca.2018.01.082.	Radiological assessment
