# Supplementary material for: Economic Evaluation of Oral Cancer Screening Programs: Review of Outcomes and Study Designs
Source: Healthcare (Basel). 2023 Apr 21;11(8):1198. doi: 10.3390/healthcare11081198 (PMC10138408; doi:10.3390/healthcare11081198)
Supplement: Supplementary file 1 [file healthcare-11-01198-s001.zip › Supplementary Material Table S3.pdf]

**Supplementary Material Table S3: Assessment using Quality of Health Economic Studies (QHES)**

| ITEMS | | WEIGHT | Dedhia 2011 | Huang 2019 | Kumdee 2018 | Speight 2006 | Subram-aniam 2009 | Meij 2002 |
|---|---|---|---|---|---|---|---|---|
| **1** | Was the study objective presented in a clear, specific, and measurable manner? | 7 | Y | Y | Y | Y | Y | Y |
| **2** | Were the perspective of the analysis (societal, third-party payer, and so on) and reasons for its selection stated? | 4 | Y | Y | Y | Y | Y | N |
| **3** | Were variable estimates used in the analysis from the best available source (i.e., Randomized Control Trial-Best, Expert Opinion-Worst)? | 8 | Y | Y | Y | Y | Y | Y |
| **4** | If estimates came from a subgroup analysis, were the groups pre-specified at the beginning of the study? | 1 | N | Y | Y | Y | Y | Y |
| **5** | Was uncertainty handled by: (i) statistical analysis to address random events; (ii) sensitivity analysis to cover a range of assumptions? | 9 | Y | Y | Y | Y | Y | Y |
| **6** | Was incremental analysis performed between alternatives for resources and costs? | 6 | Y | Y | Y | Y | Y | Y |
| **7** | Was the methodology for data abstraction (including value health states and other benefits) stated? | 5 | Y | Y | Y | Y | Y | Y |
| **8** | Did the analytic horizon allow time for all relevant and important outcomes? Were benefits and costs that went beyond 1 year discounted (3–5%), justification given for discount rate? | 7 | Y | N | Y | Y | N | N |
| **9** | Was the measurement of costs appropriate and the methodology for the estimation of quantities and unit costs clearly described? | 8 | Y | Y | Y | Y | Y | N |
| **10** | Was the primary outcome measure(s) for the economic evaluation clearly stated and were the major short term, long term and negative outcomes included? | 6 | Y | Y | Y | Y | Y | Y |
| **11** | Were the health outcomes measures/ scales valid and reliable? If previously tested valid and reliable measures were not available, was justification given for the measures/scales used? | 7 | Y | Y | Y | Y | Y | Y |
| **12** | Were the economic model (including structure), study methods and analysis, and the components of the numerator and denominator displayed in a clear transparent manner? | 8 | Y | Y | Y | Y | Y | Y |
| **13** | Were the choice of economic model, main assumptions and limitations of the study stated and justified? | 7 | Y | Y | Y | Y | Y | Y |
| **14** | Did the author(s) explicitly discuss direction and magnitude of potential biases? | 6 | Y | Y | Y | Y | Y | Y |
| **15** | Were the conclusions/recommendations of the study justified and based on the study results? | 8 | Y | Y | Y | Y | Y | Y |
| **16** | Was there a statement disclosing the source of funding for the study? | 3 | Y | Y | Y | Y | Y | N |
| | **TOTAL** | **100** | **99** | **93** | **100** | **100** | **93** | **78** |

**Instrument**
