# Supplementary material for: Economic Evaluation of Oral Cancer Screening Programs: Review of Outcomes and Study Designs
Source: Healthcare (Basel). 2023 Apr 21;11(8):1198. doi: 10.3390/healthcare11081198 (PMC10138408; doi:10.3390/healthcare11081198)
Supplement: Supplementary file 1 [file healthcare-11-01198-s001.zip › Supplementary Material Table S4.pdf]

Supplementary Material Table S4: Assessment using Philips criteria for model-based economic evaluations

STRUCTURE			Dedhia 2011	Kumdee 2018	Speight 2006	Meij 2002
S1	1	Is there a clear statement of the decision problem?	Y	Y	Y	Y
	2	Is the objective of the model specified and consistent with the stated decision problem?	Y	Y	Y	Y
	3	Is the primary decision-maker specified?	N	Y	Y	N
S2	4	Is the perspective of the model stated clearly?	Y	Y	Y	N
	5	Are the model inputs consistent with the stated perspective?	N	Y	Y	NA
S3	6	Is the structure of the model consistent with a coherent theory of the health condition under evaluation?	Y	Y	Y	Y
	7	Are the sources of the data used to develop the structure of the model specified?	Y	Y	Y	Y
S4	8	Are the structural assumptions reasonable given the overall objective, perspective and scope of the model?	Y	Y	Y	Y
S5	9	Is there a clear definition of the options under evaluation?	Y	Y	Y	Y
	10	Have all feasible and practical options been evaluated?	Y	Y	Y	Y
	11	Is there justification for the exclusion of feasible options?	Y	Y	Y	Y
S6	12	Is the chosen model type appropriate given the decision problem and specified casual relationships within the model?	Y	Y	Y	Y
S7	13	Is the time horizon of the model sufficient to reflect all important differences between the options?	Y	Y	Y	N
S8	14	Do the disease states (state transition model) or the pathways (decision tree model) reflect the underlying biological process of the disease in question and the impact of interventions?	Y	Y	Y	?
S9	15	Is the cycle length defined and justified in terms of the natural history of disease?	Y	Y	Y	N
D1	16	Are the data identification methods transparent and appropriate given the objectives of the model?	Y	Y	Y	Y
	17	Where choices have been made between data sources, are these justified appropriately?	Y	Y	Y	NA
	18	Where expert opinion has been used are the methods described and justified?	N	Y	Y	?
D2A	19	Is the choice of baseline data described and justified?	Y	Y	Y	Y
	20	Are transition probabilities calculated appropriately?	Y	Y	Y	Y
	21	Has a half-cycle correction been applied to both costs and outcomes?	N	N	N	N
	22	If not, has the omission been justified?	N	N	N	N
D2B	23	Have the methods and assumptions used to extrapolate short-term results to final outcomes been documented and justified?	NA	NA	NA	Y
	24	Are the costs incorporated into the model justified?	N	Y	Y	?
	25	Has the source for all costs been described?	Y	Y	Y	Y
	26	Have discount rates been described and justified given the target decision maker?	Y	Y	Y	N
D2C	27	Are the utilities incorporated into the model appropriate?	Y	Y	Y	Y
	28	Is the source of utility weights referenced?	Y	Y	Y	Y
D3	29	If data have been incorporated as distributions, has the choice of distributions for each parameter been described and justified?	NA	NA	Y	NA
D4D	30	If data are incorporated as point estimates, are the ranges used for sensitivity analysis stated clearly and justified?	Y	Y	Y	Y
D4C	31	Has heterogeneity been dealt with by running the model separately for different subgroups?	N	N	Y	N
C2	32	Have the results been compared with those of previous models and any differences in results explained?	Y	Y	Y	Y
